# Investigation of the relationship between the triglyceride-glucose index and metabolic and hematological parameters in obese individuals

**DOI:** 10.55730/1300-0144.6019

**Published:** 2025-04-12

**Authors:** Özge BAŞ AKSU, Feride ÖZKARA, Rıfat EMRAL, Volkan GENÇ, Demet ÇORAPÇIOĞLU, Mustafa ŞAHİN

**Affiliations:** 1 Department of Endocrinology and Metabolism, Faculty of Medicine, Ankara University, Ankara, Turkiye; 2 Department of General Surgery, Faculty of Medicine, Ankara University, Ankara, Turkiye

**Keywords:** Obesity, TyG index, AIP, FIB4, diabetes mellitus

## Abstract

**Background/aim:**

This research examined the association between the triglyceride-glucose (TyG) index and metabolic, hepatic, and hematologic variables in a population of severely obese subjects, with a focus on the assessment of glycemic control.

**Materials and methods:**

This retrospective study included 315 adult patients with a body mass index (BMI) ≥ 35 kg/m^2^ who were evaluated for bariatric surgery. Participants were grouped according to HbA1c levels: those with HbA1c ≥ 6.5% and those with HbA1c < 6.5%. TyG, atherogenic index of plasma (AIP), fibrosis-4 (FIB-4), aspartate aminotransferase-to-platelet ratio index (APRI), and hematological inflammation markers were assessed and compared between groups. To investigate associations, correlation and regression analyses were conducted.

**Results:**

Individuals with HbA1c ≥ 6.5% had significantly higher TyG, AIP, APRI, and FIB-4 values than those with lower HbA1c. The TyG index showed a strong correlation with both HbA1c (r = 0.403, p < 0.001) and AIP (r = 0.866, p < 0.001), and was identified as an independent predictor of HbA1c, explaining 20.5% of its variance. Moderate correlations were also found between liver fibrosis scores and systemic inflammatory indices. No significant differences were observed in hematologic markers across HbA1c groups, suggesting a closer link between inflammation and obesity rather than glycemic status.

**Conclusion:**

The TyG index may serve as a practical and accessible marker for metabolic risk and glycemic control in obese individuals. Its integration with other noninvasive indices like AIP, APRI, and FIB-4 may support a more comprehensive evaluation of cardiometabolic and hepatic risk profiles in clinical settings.

## 1. Introduction

In 2016, according to the World Health Organization (WHO), nearly two billion adults worldwide were affected by overweight. Additionally, approximately 650 million adults experienced obesity [1]. Healthcare systems worldwide face significant challenges, primarily due to the prevalence of obesity. This condition increases the risk of metabolic and cardiovascular diseases [2–4].

Accurately assessing comorbidity risks and tailoring interventions accordingly is one of the primary challenges in managing obesity [5,6]. While established markers like body mass index (BMI) offer a broad idea of obesity, they fail to encompass the metabolic and inflammatory factors that greatly influence the severity and prognosis of the disease [7]. The current lack of comprehensive risk assessment tools that integrate metabolic and hematological parameters poses limitations in accurately predicting outcomes and tailoring treatment approaches for obese patients.

The triglyceride-glucose (TyG) index has generated noteworthy interest as a prominent marker for metabolic risk. It is strongly correlated with insulin resistance, which is a key factor in obesity-related complications [5,8]. Unlike traditional markers, such as homeostatic model assessment of insulin resistance (HOMA-IR) or hemoglobin A1c (HbA1c), which primarily reflect insulin resistance or long-term glycemic control, the TyG index provides a more integrated assessment of both lipid and glucose metabolism [9–11]. Research has demonstrated that the TyG index is as effective as HOMA-IR in predicting insulin resistance, particularly in obese and diabetic populations [12]. The TyG index has demonstrated correlations with various clinical conditions, including metabolic syndrome, liver disease, and cardiovascular events [11,13,14].

Despite the growing body of literature on the TyG index, there remains a gap in understanding how it correlates with hematological parameters, which may further refine risk stratification in obese patients. Previous studies have focused primarily on its metabolic implications, but a more comprehensive approach that includes hematological markers may provide a deeper understanding of the systemic effects of obesity. This research endeavors to examine the correlation between the TyG index and several metabolic and hematological indicators among individuals with obesity, thus bridging a gap in current knowledge. By examining these associations, we aim to enhance risk assessment and support clinical decision-making.

## 2. Materials and methods

In this study, data from patients who were evaluated for bariatric surgery at our outpatient clinic were retrospectively reviewed. A total of 315 obese individuals who presented between January 2019 and December 2023 were included. All participants underwent a comprehensive endocrinological and metabolic evaluation as part of the standard preoperative assessment process.

Patients with a BMI ≥ 40 kg/m^2^ or a BMI ≥ 35 kg/m^2^ accompanied by at least one obesity-related comorbidity (e.g., type 2 diabetes, dyslipidemia, or hypertension) were eligible for inclusion. Individuals with a history of alcohol dependence, those using exogenous glucocorticoids, or those taking medications that could interfere with dexamethasone suppression test (DST) results were excluded. Pregnant women, individuals with hematological or solid malignancies, those with acute or chronic infections, or those with chronic inflammatory diseases were also excluded from the study.

The study protocol was approved by the Institutional Ethics Committee of Ankara University, and all procedures were conducted in accordance with the principles of the Declaration of Helsinki.

Demographic characteristics, including age, sex, weight, and height, were retrieved from patient records. BMI was calculated using the standard formula (weight in kilograms divided by the square of height in meters). All participants underwent a comprehensive metabolic and endocrine assessment as part of routine preoperative evaluation.

Laboratory parameters included fasting plasma glucose (FPG), triglycerides (TG), high-density lipoprotein cholesterol (HDL-C), low-density lipoprotein cholesterol (LDL-C), aspartate aminotransferase (AST), alanine aminotransferase (ALT), thyroid-stimulating hormone (TSH), 25-hydroxyvitamin D (25-OH-D), and glycated hemoglobin A1c (HbA1c). Following a 12 h overnight fast, blood samples were drawn.

Several indices were calculated based on these laboratory values:

- **Triglyceride-glucose index (TyG):** ln(TG [mg/dL] × FPG [mg/dL] / 2)
- **Fibrosis-4 index (FIB-4):** (age × AST) / (platelet count × √ALT)
- **Aspartate aminotransferase-to-platelet ratio index (APRI):** (AST / upper limit of normal [ULN] × 100) / platelet count, where the ULN for AST was 35 U/L
- **Atherogenic index of plasma (AIP):** log10(TG / HDL-C)
  Hematological parameters, including neutrophil, lymphocyte, monocyte, and platelet counts, were measured using an automated hematology analyzer.

The following inflammatory indices were calculated:

- **Pan-immune-inflammation value (PIV):** neutrophils × platelets × monocytes / lymphocytes
- **Systemic immune-inflammation index (SII):** neutrophils × platelets / lymphocytes
- **Monocyte-to-HDL-C ratio (MHR):** monocytes / HDL-C
- **Neutrophil-to-lymphocyte ratio (NLR):** neutrophils / lymphocytes

### 2.1. Statistical analysis

All statistical analyses were performed using IBM SPSS Statistics for Windows, version 22.0 (IBM Corp., Armonk, NY, USA). The normality of continuous variables was assessed using the Shapiro–Wilk test. Descriptive statistics were reported as counts and percentages (%) for categorical data. For continuous variables, normally distributed data were expressed as mean ± standard deviation (SD), while nonnormally distributed data were presented as median (minimum–maximum).

To compare categorical variables between groups, the chi-square (χ^2^) test was applied. The choice of test for continuous variables was based on their distribution: the independent samples t-test was used for normally distributed variables, and the Mann–Whitney U test for those not following a normal distribution.

Spearman correlation analysis was employed to explore associations between variables. Additionally, stepwise multiple regression analysis was conducted to identify independent predictors of HbA1c levels. A p-value of <0.05 was accepted as the threshold for statistical significance, with results interpreted within a 95% confidence interval.

## 3. Results

A total of 315 participants were included in the study. The basic demographic and laboratory characteristics of the participants according to HbA1c levels are presented in Table 1. The study population consisted of 51.1% (n = 161) females and 48.9% (n = 154) males. When participants were divided into two groups according to HbA1c levels, there were 44 individuals (14%) in the HbA1c ≥ 6.5% group and 271 individuals (86%) in the HbA1c < 6.5% group. Significant differences were observed between the two groups in several key parameters. FPG levels were significantly higher in the HbA1c ≥ 6.5% group (126 mg/dL vs 93 mg/dL, p < 0.001). When liver function tests were examined, AST and ALT values were significantly higher in the HbA1c ≥ 6.5% group (p = 0.018 and p = 0.014, respectively). Similarly, triglyceride levels were significantly higher in the HbA1c ≥ 6.5% group (173 mg/dL vs 132 mg/dL, p = 0.014). There was no significant difference between the groups in terms of HDL-C and LDL-C levels.

When liver fibrosis and inflammation markers were evaluated, FIB-4 score, APRI score, and AIP values were found to be statistically significantly higher in the HbA1c ≥ 6.5% group (p = 0.002, p = 0.033, and p = 0.002, respectively). The mean TyG index was significantly higher in the HbA1c ≥ 6.5% group (5.01 ± 0.27 vs 4.70 ± 0.26, p < 0.001).

No statistically significant differences were observed between the groups regarding leukocyte count, neutrophil count, monocyte count, lymphocyte count, platelet count, MHR, NLR, SII, and PIV values.

The results of the correlation analysis between variables are presented in Table 2. The parameter that showed the strongest correlation with HbA1c was the TyG index (r = 0.403, p < 0.001). HbA1c also showed positive correlations with AIP (r = 0.260, p < 0.001), FIB-4 (r = 0.197, p < 0.001), MHR (r = 0.154, p < 0.01), and APRI (r = 0.150, p < 0.01). The TyG index exhibited the strongest correlation with AIP (r = 0.866, p < 0.001).

The results of the stepwise regression analysis to determine the independent factors affecting HbA1c levels are shown in Table 3. Based on previous findings, age, TyG index, FIB-4 score, APRI score, MHR, HDL, and LDL, which were shown to be associated with HbA1c, were identified as independent variables (APRI was excluded from the analysis due to multicollinearity). The TyG index was identified as the strongest independent variable explaining 20.5% of the variance in HbA1c (p < 0.001). When age was added to the model, the explained variance increased to 24% (p < 0.001). The regression model equation was formulated as follows: HbA1c = −1.094 + (1.359 × TyG index) + (0.015 × age).

## 4. Discussion

This study investigated the associations between the TyG index and various metabolic and inflammatory parameters in obese individuals, including glycemic regulation, lipid profile, liver function, and hematological indices. Participants with HbA1c levels ≥ 6.5% demonstrated significantly elevated TyG, AIP, APRI, and FIB-4 scores. Notably, TyG showed strong correlations with HbA1c and AIP, and regression analysis revealed TyG as an independent predictor of HbA1c, accounting for 20.5% of its variance. These findings suggest that TyG may serve as an indirect yet useful indicator of glycemic regulation and could be valuable in assessing metabolic risk in obese populations.

Previous studies have consistently demonstrated a robust correlation between the TyG index and established markers of insulin resistance, such as HOMA-IR [15–18]. In research settings where direct insulin measurements are unavailable—particularly in retrospective studies—TyG serves as a practical surrogate due to its simplicity and accessibility. While HbA1c is commonly used in clinical practice to monitor glycemic control and diagnose diabetes, its relatively high cost may limit its use in some settings [19]. In contrast, the TyG index provides an affordable and widely available alternative. Supporting this, Babic et al. found that patients with type 2 diabetes and HbA1c ≥ 7% had significantly higher TyG scores compared to those with HbA1c < 7% (5.21 ± 0.04 vs 4.94 ± 0.05, p < 0.001), in line with our findings [20].

Interestingly, no significant association was identified between TyG and BMI in our analysis. This may be attributed to BMI’s inherent limitation of reflecting total body mass without providing detailed insights into fat distribution or metabolic activity [21, 22]. While BMI remains a widely used indicator for obesity classification, it may not fully capture the underlying metabolic burden. In contrast, the TyG index—derived from routine biochemical parameters—has the potential to more accurately reflect early metabolic disturbances, particularly those related to insulin resistance. This feature positions TyG as a valuable complement to HbA1c, especially in the context of obesity-related risk screening. Furthermore, in healthcare settings with restricted access to extensive laboratory infrastructure, TyG offers a feasible, low-cost alternative for metabolic evaluation.

The burden of obesity extends to multiple organ systems, serving as a critical risk factor for metabolic conditions such as insulin resistance, type 2 diabetes, cardiovascular diseases, and liver disease [3,23–26]. Identifying the metabolic burden in obese individuals at an early stage is essential for guiding clinical decision-making and personalizing therapeutic strategies. Within this context, practical and inexpensive biomarkers such as the TyG and AIP indices have gained increasing attention for their utility in assessing metabolic health.

The TyG index, which incorporates fasting glucose and triglyceride concentrations, provides indirect insight into both insulin resistance and glycemic status. In contrast, AIP is based on the logarithmic ratio of triglycerides to HDL-C and is primarily used to evaluate atherogenic dyslipidemia, making it a relevant marker for cardiovascular risk stratification [27]. Despite sharing triglycerides as a common component, the differences in their mathematical formulations reflect distinct aspects of metabolic imbalance—TyG being more sensitive to glycemic dysfunction and AIP more aligned with lipid abnormalities and cardiovascular risk.

Several studies have highlighted the clinical utility of these indices. Zhu et al. found that AIP demonstrated a stronger association with obesity than conventional lipid measures [28]. Furthermore, recent research has suggested that AIP could serve as a dependable predictor for the development of T2DM [29]. In line with these observations, our study revealed that both TyG and AIP values were significantly elevated in individuals with HbA1c ≥ 6.5%, reinforcing the notion that disturbances in glucose metabolism and dyslipidemia frequently coexist. The combined use of TyG and AIP may thus enhance metabolic risk assessment by offering a broader view of the underlying pathophysiology.

Beyond evaluating individual indices, integrating multiple metabolic markers may provide a more robust estimation of cardiometabolic risk. In a study conducted by Si et al., it was demonstrated that a combined assessment of the TyG, AIP, and MHR indices offered superior predictive value for identifying subclinical coronary artery disease compared to using these markers independently [30]. This supports the perspective that the multifaceted nature of metabolic syndrome is best understood through a multidimensional approach.

Each of these indices captures different physiological domains: TyG reflects insulin resistance and glycemic regulation, AIP represents lipid imbalance, and MHR is associated with systemic inflammation and vascular health. When interpreted collectively, these markers may offer a more complete evaluation of an individual’s metabolic status. This approach aligns with current clinical efforts to move beyond single-metric evaluations and toward more comprehensive, integrated risk stratification models in populations with obesity or metabolic dysfunction.

Among the metabolic complications of obesity, liver-related changes—including structural and functional alterations—are particularly noteworthy. The shift from the term “nonalcoholic fatty liver disease” (NAFLD) to “metabolic dysfunction-associated fatty liver disease” (MAFLD) highlights the central role of metabolic derangements in hepatic steatosis and fibrosis [31,32]. The increasing prevalence of MAFLD mirrors global obesity trends and has made it one of the leading causes of chronic liver disease. Mechanistically, hepatic lipid accumulation and fibrosis are driven by insulin resistance, lipotoxicity, and persistent low-grade inflammation [33].

Given the limitations of liver biopsy—its invasiveness, cost, and limited accessibility—noninvasive scoring systems such as FIB-4 and APRI have gained popularity for the evaluation of hepatic fibrosis. In our study, these indices were significantly elevated among individuals with HbA1c ≥ 6.5%, suggesting a link between impaired glycemic control and liver involvement. Similarly, prior research has shown that combining TyG with gamma-glutamyl transferase enhances the diagnostic accuracy for nonalcoholic steatohepatitis (NASH) in severely obese patients [34].

In our analysis, no statistically significant differences were observed in hematological inflammatory markers when participants were grouped according to HbA1c levels. This finding may imply that systemic inflammation is more closely associated with obesity-related mechanisms than with glycemic control alone. Chronic low-grade inflammation in obesity is known to influence metabolically active organs—such as adipose tissue, liver, and skeletal muscle—via cytokine-mediated pathways and cellular stress responses [35].

Interestingly, we observed a moderate correlation between the TyG index and MHR, a marker that reflects both metabolic and inflammatory components and has been previously linked to cardiometabolic risk [30]. This association may be attributed to the inclusion of HDL-C in MHR, which has established antiinflammatory and antioxidant roles and is also involved in the regulation of insulin sensitivity. Given that TyG is a surrogate marker of insulin resistance derived from triglyceride and glucose levels, the inverse role of HDL-C in MHR may reveal a shared metabolic background, explaining the link between the two indices.

In contrast, no meaningful associations were found between TyG and indices such as SII, PIV, or NLR, which are primarily constructed from immune cell counts and reflect broader systemic inflammation. These indices focus on innate immune responses and do not directly represent the metabolic dysfunctions central to insulin resistance. Therefore, the stronger association between TyG and MHR likely reflects their mutual relation to lipid-glucose metabolism, whereas SII and PIV may operate through partially distinct inflammatory pathways.

Although several studies have highlighted the potential of these indices in evaluating conditions ranging from diabetes to cardiovascular disease and cancer, their reliability can be influenced by a wide range of clinical factors [36,37]. For instance, analyses using NHANES data have linked high SII levels to increased diabetes risk and poorer prognosis [38]. However, the variability of complete blood count-derived markers and their susceptibility to nonmetabolic confounders can contribute to inconsistent results across studies. In our cohort, the absence of significant differences in these indices further suggests that their relationship to glycemic regulation may be limited, and their utility might be more pronounced in settings where inflammatory sources are more clearly defined.

Furthermore, we found moderate correlations between APRI, FIB-4, and systemic inflammatory indices such as SII and PIV. While this may imply a potential link between hepatic fibrosis and systemic inflammation, it is also possible that these findings reflect random associations influenced by overlapping nonspecific hematologic parameters. Given the lack of consistent associations between these inflammatory indices and glycemic or metabolic markers, the clinical value of such correlations remains uncertain. These results highlight the need for cautious interpretation of hematologic indices and underscore the importance of validating their relevance in larger and more targeted populations.

This study is subject to several limitations that should be acknowledged. Being a single-center investigation with a relatively small sample size, the generalizability of the findings is inherently restricted. The study population consisted exclusively of severely obese individuals undergoing evaluation for bariatric surgery, which limits the applicability of the results to individuals with a BMI below 35 kg/m^2^. Moreover, due to the retrospective nature of the data, we were unable to include direct measures of insulin resistance such as fasting insulin or HOMA-IR. Instead, HbA1c was used as a surrogate marker for glycemic control. Although the TyG index was significantly associated with HbA1c, the lack of direct insulin resistance metrics limits definitive conclusions regarding this relationship. Nonetheless, previous studies have demonstrated this association, and our results are consistent with existing evidence.

Additionally, comorbidities such as hypertension and dyslipidemia were not systematically evaluated, which may have influenced the metabolic profiles observed. Waist circumference, a key anthropometric indicator in defining obesity and metabolic syndrome, was not consistently available and therefore could not be analyzed. Furthermore, our assessment of hematologic markers relied solely on complete blood count data, and more specific indicators of inflammation or oxidative stress were not included.

Despite these limitations, our study offers a notable strength in its multidimensional approach. By concurrently evaluating glycemic, lipid, hepatic, and inflammatory parameters within the same cohort, we provide a broader perspective on metabolic risk in obese individuals. Importantly, we highlight the potential clinical relevance of inexpensive and noninvasive markers such as TyG, AIP, APRI, and FIB-4. These indices may assist in identifying high-risk individuals and tailoring early intervention strategies. Future research involving larger, prospective cohorts will be essential to confirm and expand upon these findings.

## Figures and Tables

**Table 1:** Basic demographic and laboratory characteristics of the study population according to the HbA1c level.

	HbA1c <6.5 (n=271)	HbA1c ≥ 6.5 (n=44)	Total (n=315)	p
Sex, F/M	134/137	27/17	161/154	0.142*
	**Median (min–max)**	**Median (min–max)**	**Median (min–max)**	
Age, years	35 (18–65)	48 (20–67)	37 (18–67)	**<0.001** **
BMI, kg/m^2^	42.87 (30.10–71.18)	45.84 (36.33–77.19)	42.87 (30.10–77.19)	0.071**
FPG, mg/dL	93 (66–186)	126 (80–374)	94 (66–374)	**<0.001** **
AST, U/L	20 (11–83)	23 (10–115)	21 (10–115)	**0.018** **
ALT, U/L	23 (7–155)	32 (10–132)	25 (7–155)	**0.014** **
TG, mg/dL	132 (36–556)	173 (61–355)	139 (36–556)	**0.014** **
HDL-C, mg/dL	44 (27–78)	43 (28–75)	44 (27–78)	0.636**
LDL-C, mg/dL	112 (37–222)	116 (64–177)	112 (37–222)	0.461**
TSH, mIU/mL	2.17 (0.01–21.90)	1.81 (0.69–8.45)	2.16 (0.01–21.90)	0.069**
25-OH-D level, μg/L	10.88 (5–54)	10.88 (4.00–41.30)	10.88 (4–54)	0.236**
1 mg DST, μg/dL	0.58 (0.20–2.80)	0.59 (0.31–1.75)	0.58 (0.20–2.80)	0.213**
FIB-4 score	0.48 (0.10–2.39)	0.63 (0.27–2.32)	0.50 (0.11–2.39)	**0.002** **
APRI score	0.20 (0.06–0.93)	0.27 (0.78–1.24)	0.20 (0.06–1.24)	**0.033** **
AIP	1.06 (–0.32 to 2.75)	1.27 (0.11–2.40)	1.14 (–0.32 to 2.75)	**0.002** **
Leukocyte count (10^9^/L)	8.52 (4.41–19.51)	8.18 (4.83–13.65)	8.52 (4.41–19.51)	0.925**
Neutrophil count (10^9^/L)	5.12 (1.53–13.41)	4.90 (2.58–8.68)	5.09 (1.53–13.41)	0.642**
Monocyte count (10^9^/L)	0.60 (0.16–1.85)	0.60 (0.25–1.30)	0.60 (0.16–1.85)	0.877**
Lymphocyte count (10^9^/L)	2.58 (0.22–5.98)	2.36 (1.23–5.64)	2.54 (0.22–5.98)	0.430**
PLT (10^9^/L)	298 (121–692)	297 (144–516)	298 (121–692)	0.997**
MHR	0.01 (0.00–0.03)	0.01 (0.04–0.39)	0.01 (0.00–0.39)	0.853**
NLR	1.94 (0.66–27.09)	2.08 (1.08–5.93)	1.95 (0.66–27.09)	0.610**
SII	618.88 (135.49–10132.00)	590.32 (280.15–1458.00)	612.41 (135.49–10132.00)	0.430**
PIV	359.04 (51.48–6991.08)	351.37 (96.61–1179.89)	359.04 (51.48–6991.08)	0.889**
	**Mean ± SD**	**Mean ± SD**	**Mean ± SD**	
HbA1c, %	5.68±0.32	7.50±1.63	5.93±0.92	**<0.001** ***
TyG Index	4.70±0.26	5.01±0.27	4.74±0.28	**<0.001** ***

* Chi-square test,

** Mann–Whitney U test,

*** Independent t-test.

Bold values indicate statistically significant results.

F: Female, M: Male, BMI: Body mass index, FPG: Fasting plasma glucose, AST: Aspartate Aminotransferase, ALT: Alanine aminotransferase, TG: Triglyceride, HDL-C: High-density lipoprotein cholesterol, LDL: Low-density lipoprotein cholesterol, TSH: Thyroid-stimulating hormone, HbA1c: Glycated hemoglobin A1c, DST: Dexamethasone suppression test, TyG: Triglyceride-Glucose Index, FIB-4: Fibrosis-4 Index, APRI: Aspartate Aminotransferase to Platelet Ratio Index, AIP: Atherogenic Index of Plasma, PLT: Platelet, MHR: Monocyte-to-HDL-C ratio, NLR: Neutrophil–lymphocyte ratio, SII: Systemic inflammation index, PIV: Panimmune inflammation value.

**Table 2:** Correlation among variables.

		1	2	3	4	5	6	7	8
**1**.	**BMI**								
**2**.	**HbA1c**	0.05							
**3**.	**TyG Index**	0.08	0.403***						
**4**.	**FIB-4**	−0.01	0.197***	0.122*					
**5**.	**APRI**	−0.05	0.150**	0.242***	0.588***				
**6**.	**AIP**	0.122*	0.260***	0.866***	−0.01	0.203***			
**7**.	**MHR**	0.05	0.154**	0.349***	−0.244***	0.02	0.571***		
**8**.	**SII**	0.03	−0.01	−0.06	0.411***	0.440***	−0.06	−0.01	
**9**.	**PIV**	0.01	0.07	0.11	0.428***	0.389***	0.118*	0.422***	0.824***

n = 315

* p < 0.05

** p < 0.01

*** p < 0.001

BMI: Body mass index, HbA1c: Glycated hemoglobin A1c, TyG: Triglyceride-Glucose Index, FIB-4: Fibrosis-4 Index, APRI: Aspartate Aminotransferase to Platelet Ratio Index, AIP: Atherogenic Index of Plasma, MHR: Monocyte-to-HDL-C ratio, SII: Systemic Inflammation Index, PIV: Panimmune inflammation value

**Table 3:** Stepwise regression analysis of independent predictors of HbA1c.

Model		Unstandardized coefficients		Standardized coefficients	t	Significance	Change statistics			Collinearity statistics		Durbin-Watson
		B	Std. error	Beta			R square change	F change	Significance	Tolerance	VIF	
1	Constant	−0.931	0.767		−1.215	0.225						1.574
	TyG Index	1.447	0.161	0.453	8.978	<0.001	0.205	80.609	<0.001	1.000	1.000	
2	Constant	−1.094	0.752		−1.455	0.147						
	TyG Index	1.359	0.160	0.425	8.519	<0.001	0.035	14.320	<0.001	0.979	1.022	
	Age	0.015	0.004	0.189	3.784	<0.001				0.979	1.022	
